# Triage and risk classification protocols in Pediatric emergency

**DOI:** 10.1016/j.rppede.2016.06.005

**Published:** 2016

**Authors:** Emílio Carlos Elias Baracat

**Affiliations:** a Departamento de Pediatria, Faculdade de Ciências Médicas, Universidade Estadual de Campinas (Unicamp), Campinas, SP, Brazil

The use of triage protocols in urgency and emergency services is a key strategy for the rapid treatment of the patient with severe clinical condition. The urgency categorization and waiting time definition are considered quality indicators in patient care, especially in situations when there is a large volume of patients.

Emergency service triage is a relatively recent phenomenon, introduced in 1950 in the United States. Several systems have been developed since then to guide health teams to perform the correct decision-making.^1^

The discussion in the literature on risk classification tools in Pediatric emergency is an ongoing one and available tools are applied in different epidemiological situations. The majority of triage scales are stratified into five urgency levels or categories. The most often used scales in Pediatrics are the PaedCTAS (The Paediatric Canadian Triage and Acuity Scale), MTS (The Manchester Triage System), ESI (Emergency Severity Index) and ATS (Australian Triage Scale), all validated with the inclusion of basic parameters of Pediatric response in acute injuries. Among these parameters, the patient's vital data, such as respiratory rate, heart rate, level of consciousness, body temperature and oxygen saturation, in addition to the main complaint, comprise the main components.^1^^–^ 3 The PaedCTAS, MTS and ESI systems contain specific parts for the Pediatric population.^2^^,^ 4 ^,^ 5 In a study by van Veen & Moll, with a literature review, the MTS and PaedCTAS systems showed better reliability and efficacy for use in Pediatric emergency.^6^

For its validation, it is essential for the tool to be reliable and safe.^7^ That is determined by an agreement between observers (evaluation of the same patient by different professionals) and in the same observer (the same patient or scenario assessed at different times) (Kappa coefficient). This measure of agreement has a maximum value of 1 (total agreement) and can be close to zero, indicating no agreement.^8^ In studies evaluating the use of severity assessment scales, it is essential to identify and correct interobserver variability in search for a high Kappa coefficient before field use.

In this issue of Revista Paulista de Pediatria, Barbosa and colleagues propose the implementation of a new risk classification tool in Pediatric emergency – CLARIPED, to be used in the national territory.^9^ For that purpose, the study authors carefully followed the risk classification scale validation steps, with prior discussion with a group of specialists, staff training, pre-testing, adjustment and final testing, obtaining a high Kappa coefficient (0.79). Risk classification into five categories is proposed, using the markers of vital signs, reason for consultation and overall assessment of general health status, pain, fever, age and return to the service. The results showed agreement between the risk classification and the use of diagnostic and therapeutic resources.

The comparison of the study results with previously validated tools in the literature and the increase of its large-scale application in different Pediatric emergency contexts can reinforce the proposal, as well as its reliable and safe inclusion.
